# Supplementary material for: Multiple Testing, Cut-Point Optimization, and Signs of Publication Bias in Prognostic FDG–PET Imaging Studies of Head and Neck and Lung Cancer: A Review and Meta-Analysis
Source: Diagnostics (Basel). 2020 Dec 1;10(12):1030. doi: 10.3390/diagnostics10121030 (PMC7761090; doi:10.3390/diagnostics10121030)
Supplement: Supplementary file 1 [file diagnostics-10-01030-s001.pdf]

Figure S1: Forest plots for LC in HNSCC and NSCLC

A **HNSCC**

Study or Subgroup	log[Hazard Ratio]	SE	Weight	Hazard Ratio IV, Fixed, 95% CI
Schwartz 2004 (54)	2.104	1.07	0.1%	8.20 [1.01, 66.76]
Torizuka 2009 (50)	1.9257	0.713	0.2%	6.86 [1.70, 27.75]
Brun 2002 (47)	1.2238	0.5631	0.3%	3.40 [1.13, 10.25]
Allal 2004 (120)	0.9416	0.4623	0.4%	2.56 [1.04, 6.35]
Kim 2007 (52)	1.4702	0.4454	0.5%	4.35 [1.82, 10.41]
Cacicedo 2017 (58)	0.0435	0.1624	3.5%	1.04 [0.76, 1.44]
Liao 2009 (109)	0.076	0.031	95.1%	1.08 [1.02, 1.15]
**Total (95% CI)**			**100.0%**	**1.10 [1.03, 1.16]**

Heterogeneity: $Chi^2 = 27.50$, df = 6 (P = 0.0001); $I^2 = 78\%$
Test for overall effect: Z = 3.08 (P = 0.002)

Hazard Ratio
IV, Fixed, 95% CI

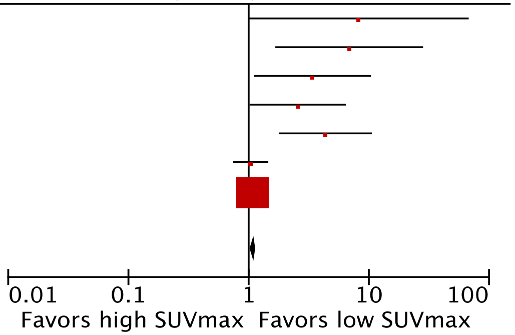

B **NSCLC**

Study or Subgroup	log[Hazard Ratio]	SE	Weight	Hazard Ratio IV, Fixed, 95% CI
Hamamoto 2011 (26)	3.248	22.8428	0.0%	25.74 [0.00, 7.152E20]
Aoki 2016 (74)	2.5619	13.892	0.0%	12.96 [0.00, 8.660E12]
Takeda 2011 (95)	2.641	0.6581	1.3%	14.03 [3.86, 50.95]
Shirai 2015 (45)	0.0768	0.0754	98.7%	1.08 [0.93, 1.25]
**Total (95% CI)**			**100.0%**	**1.12 [0.96, 1.29]**

Heterogeneity: $Chi^2 = 15.04$, df = 3 (P = 0.002); $I^2 = 80\%$
Test for overall effect: Z = 1.47 (P = 0.14)

Hazard Ratio
IV, Fixed, 95% CI

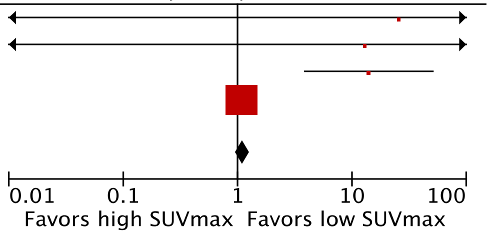

Figure S2: Eggers regression versus meta-analysis

Linear regression of log hazard ratio versus standard error of log HR. Should have slope zero in the absence of publication bias. Intercept can be interpreted as “publication bias adjusted” estimate of the effect of FDG uptake in the limit of a large study

The regression has the type:
Regression: ln(HR)~b0+b1*Std.Error(lnHR)+error

Full R code for this analysis available at https://rpubs.com/IvanVogelius/Eggers

Head and Neck Cancer

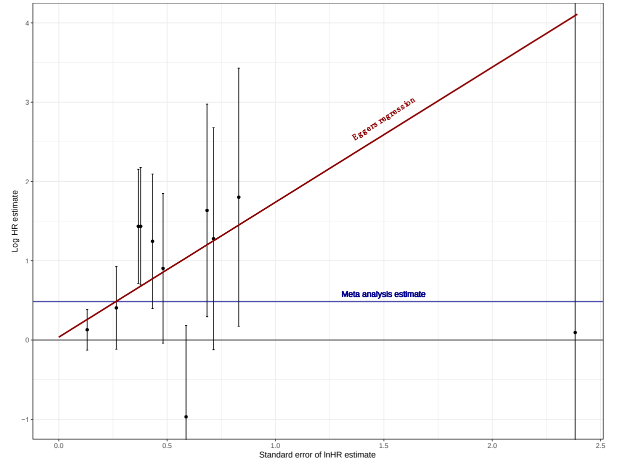

Overall survival
Coefficients with 95% confidence interval:
b0=0.037 (-0.6 to 0.66)
b1= 1.7 (-0.24 to 3.6)

p-values:
Nonzero slope: p=0.08 (publication bias)
Nonzero intercept: p=0.90 (effect of FDG)

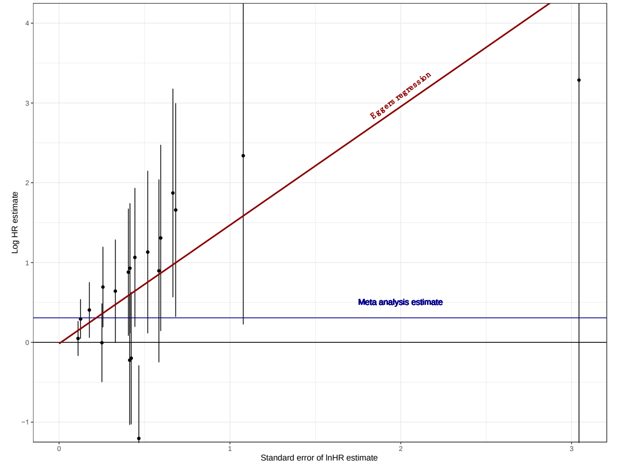

Disease free survival

Coefficients with 95% confidence interval:

b0=-0.016 (-0.34 to 0.31)

b1= 1.48 (0.28 to 2.69)

p-values:

Nonzero slope: **p=0.02** (publication bias)

Nonzero intercept: p=0.92 (effect of FDG)

Non-small cell lung cancer

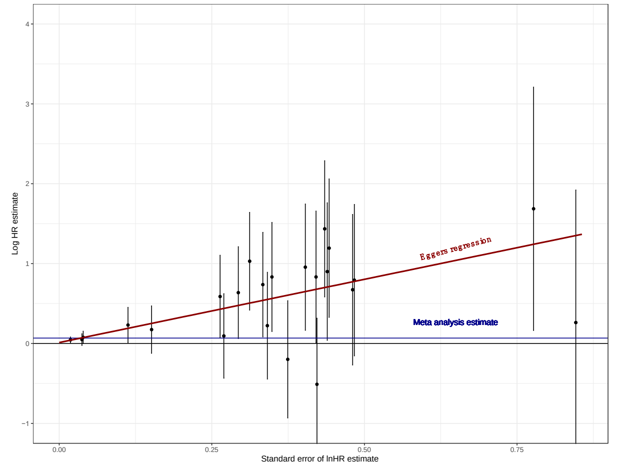

Overall survival

Coefficients with 95% confidence interval:

b0=0.012 (-0.03 to 0.05)

b1= 1.6 (1.0 to 2.2)

p-values:

Nonzero slope: **p<0.001** (publication bias)

Nonzero intercept: p=0.56 (effect of FDG)

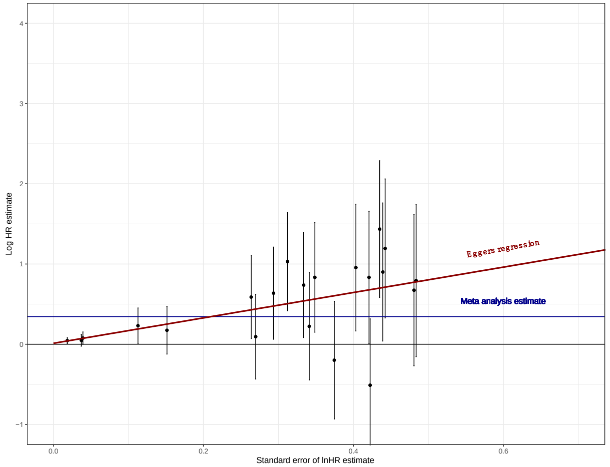

Disease free survival

Coefficients with 95% confidence interval:

b0=0.015 (-0.28 to 0.31)

b1= 1.67 (0.42 to 2.9)

p-values:

Nonzero slope: **p=0.014** (publication bias)

Nonzero intercept: p=0.91 (effect of FDG)

Table S1: Data extracted from the included studies.

	DFS		OS	
**HNSCC**	**Univariate**	**Multivariate**	**Univariate**	**Multivariate**
Akagunduz 2015	**HR 1.49 (p=0.017)***		**HR 1.5 (p=0.022)***	
Allal 2004	HR=2.5 (p=0.005)*	**HR=1.9 (1.0-3.70, p=0.051)**		
Baschnagel 2015		**HR=0.82 (0.36-1.87, p=0.64)**		
Cacicedo 2017			**HR=1.1 (p=0.972)***	
Chan 2017	**HR=0.995 (0.507-1.952, p=0.987)**			
Chung 2009	HR=1.1 (0.42-2.69, p=0.88)	**HR=0.8 (0.28-2.07, p=0.59)**		
Halfpenny 2002			HR=4.4 (1.652-11.558, p=0.003)	**HR=4.2 (1.146-8.496, p=0.002)**
Higgins 2012	**HR=3.1 (p=0.029)***			
Kim 2007		**HR=3.7 (1.06-14.63, p=0.043)**		
Kitajima 2014	**HR=1.05 (p=0.66)***			
Komar 2014			**HR=1.14 (p=0.317)***	
Koyasu 2014	**HR=2.45 (p=0.025)**			
Kunkel 2003				**HR=5.13 (1.34-19.65, p=0.017)**
Machtay 2009	HR=2.9 (p=0.007)*	**HR=2.41 (1.1-5.32, p=0.03)**	HR=2.4 (p=0.016)*	**HR=2.47 (0.98-6.26, p=0.06)**
Minn 1997			**HR=4.2 (1.6-11.0, p=0.002)**	
Moon 2015	**HR=26.754 (p=0.278)**			
Ng 2016		**HR=2.532 (1.121-5.715, p=0.025)**		**HR=3.477 (1.505-8.031, p=0.004)**
Preda 2016		**HR=10.37 (1.22-87.95, p=0.03)**		
Roh 2007	**HR=2.0 (p=0.007)***			
Schwartz 2004	**HR=1.34 (p=0.02)***			
Schwartz 2014	**HR=0.30 (p=0.01)**			
Suzuki 2014				**HR=6.06 (p<0.03)**
Suzuki-Shibata 2017		**HR=5.25 (1.39-19.89, p=0.015)**		**HR=3.59 (0.89-14.45, p=0.073)**
Torizuka 2009	**HR=6.5 (p=0.0051)***			
Xie 2010	**HR=2.9 (p=0.0163)***			
**NSCLC**				
Ahuja 1998			HR=1.9 (p=0.0049)*	**HR=1.8 (p=0.026)**

Borst 2005			**HR=4.2 (p<0.001)***	
Carvalho 2013			**HR=1.19 (p=0.25)***	
Cerfolio 2005			HR=2.5 (p<0.001)*	**HR=2.8 (2.1-6.4)**
Chen 2012	HR=1.0 (0.59-1.54)	**HR=1.2 (0.54-2.45)**		
Clarke 2012	**HR=2.4 (p=0.04)***			
Hofheinz 2016	HR=1.85 (p=0.099)*			
Horne 2014	**HR=2.79 (p=0.03)**		**HR=2.09 (p=0.027)**	
Hyun 2015	HR=1.71 (1.08-2.68, p=0.02)	**HR=1.14 (0.67-1.92, p=0.618)**	HR=1.79 (0.98-3.25, p=0.058)	**HR=0.82 (0.39-1.70, p=0.596)**
Imamura 2011	HR=1.1 (p=0.609)*		**HR=2.3 (p=0.017)***	
Jiang 2018				**HR=1.08 (0.75-1.57, p<0.05)**
Kohutek 2015			HR=2.53 (p=0.0007)	**HR=1.89 (p=0.03)**
Lee 2012			HR=1.8 (1.09-2.86)	**HR=2.6 (1.67-4.17)**
Nair 2013	**HR=1.35 (p=0.0096)***		**HR=1.05 (p=0.1906)***	
Nawara 2012			**HR=1.3 (p=0.757)***	
Pyka 2015			**HR 1.96 (p=0.161)***	
Sasaki 2005	HR=5.1 (p<0.0001)	**HR=4.3 (p=0.001)**	HR=5.6 (p=0.02)	**HR=5.4 (p=0.03)**
Shirai 2017	HR=1.32 (p=0.02)*		**HR=1.26 (p=0.04)***	
Sugawara 1999			**HR=0.6 (p=0.2256)***	
Takeda 2014	HR=6.25 (p<0.01)	**HR=5.94 (p<0.01)**	HR=4.18 (p<0.01)	**HR=2.46 (p=0.04)**
Takeda 2017	**HR=2.12 (p=0.03)***			
Ulger 2014				**HR=1.046 (p=0.015)**
Vansteenkiste 1999			HR=3.9 (p=0.001)*	**HR=3.3 (1.39-7.94, p=0.007)**
Vesselle 2007			HR=2.0 (1.15-3.5)	**HR=1.3 (0.64-2.44)**
Vu 2013	**HR=2.21 (p=0.1)**			
Xiang 2012	HR=3.3 (1.529-6.992)	**HR=2.6 (1.115-6.158)**	HR=2.7 (1.251-5.699)	**HR=2.3 (1.006-5.474)**
Yilmaz 2018	HR=1.058 (0.635-1.763, p=0.828)	**HR=1.022 (0.578-1.805, p=0.941)**	HR=1.071 (0.641-1.791, p=0.792)	**HR=1.099 (0.649-1.860, p=0.726)**

Data highlighted with bold were entered in RevMan. HR estimated from univariate log-rank test was only entered in RevMan if MVA was not performed. *HR estimated from Kaplan-Meier plot.
